# Supplementary material for: Analysis of Chromatin Accessibility and DNA Methylation to Reveal the Functions of Epigenetic Modifications in Cyprinus carpio Gonads
Source: Int J Mol Sci. 2023 Dec 25;25(1):321. doi: 10.3390/ijms25010321 (PMC10778764; doi:10.3390/ijms25010321)
Supplement: Supplementary file 1 [file ijms-25-00321-s001.zip › Supplementary Materials.pdf]

Figure S1. GO enrichment analyses of DMGs between ovaries and testes. (A) GO enrichment of promoter-DMGs. (B) GO enrichment of genebody-DMGs. Figure S2. Venn diagrams of promoter-DMGs (A) and genebody-DMGs (B) across different contexts. Table S1. Summary of sequencing quality and reads alignment statistics of common carp gonadal ATAC-seq data. Table S2. Details and annotations of peaks in common carp gonads. Table S3. List of DAGs between ovaries and testes. Table S4. List of differential TF binding motifs between ovaries and testes ($p \leq 0.01$). Table S5. List of the overlaps between DAGs and DEGs. Table S6. Summary of gonadal BS-seq data. Table S7. Statistics of gonadal genome-wide methylation in different contexts. Table S8. DMRs between ovaries and testes in CG contexts. Table S9. List of DMGs in CG contexts. Table S10. List of DMGs in CHG contexts. Table S11. List of DMGs in CHH contexts. Table S12. Overlaps between promoter-DMRs and DEGs. Table S13. Overlaps between genebody-DMRs and DEGs.
