# Supplementary figures and images for: Analysis of Chromatin Accessibility and DNA Methylation to Reveal the Functions of Epigenetic Modifications in Cyprinus carpio Gonads
Source: Int J Mol Sci. 2023 Dec 25;25(1):321. doi: 10.3390/ijms25010321 (PMC10778764; doi:10.3390/ijms25010321)

## Slide 1
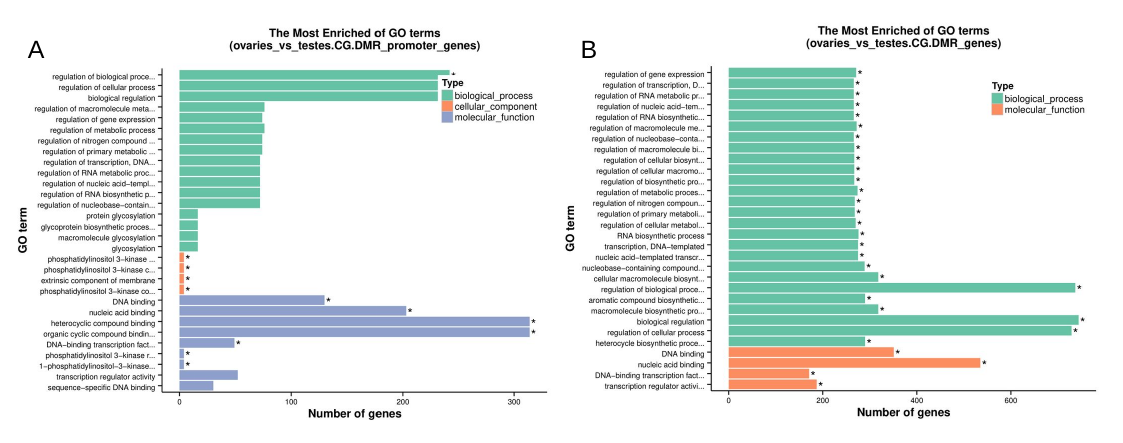

A
B

Supplement: Supplementary file 1 [file ijms-25-00321-s001.zip › Figure S1. GO enrichment analyses of DMGs between ovaries and testes.pptx]

## Slide 1
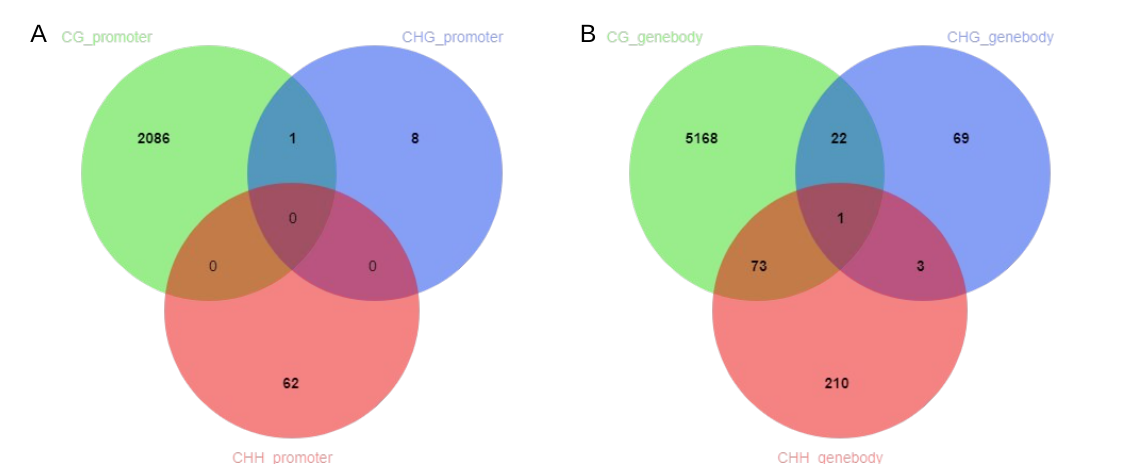

A
B

Supplement: Supplementary file 1 [file ijms-25-00321-s001.zip › Figure S2. Venn diagrams of promoter-DMGs (A) and genebody-DMGs (B) across different contexts.pptx]
